# Intralymphatic immunotherapy with birch and grass pollen extracts. A randomized double‐blind placebo‐controlled clinical trial

**DOI:** 10.1111/cea.14307

**Published:** 2023-04-04

**Authors:** Lars Ahlbeck, Emelie Ahlberg, Linn Stuivers, Janne Björkander, Ulla Nyström, Pavlos Retsas, Dhanapal Govindaraj, Maria C. Jenmalm, Karel Duchén

**Affiliations:** ^1^ Allergy Center University Hospital Linköping Sweden; ^2^ Division of Inflammation and Infection, Department of Biomedical and Clinical Sciences Linköping University Linköping Sweden; ^3^ Futurum Academy of Health and Care Jönköping Sweden; ^4^ Department of Biomedical and Clinical Sciences, Division of Children's and Women's Health Linköping University Linköping Sweden

**Keywords:** allergy, hypersensitivity, intralymphatic immunotherapy, rhinoconjunctivitis

## Abstract

**Introduction:**

There is a need to evaluate the safety and efficacy of intralymphatic immunotherapy (ILIT) for inducing tolerance in patients with allergic rhinitis.

**Methods:**

Thirty‐seven patients with seasonal allergic symptoms to birch and grass pollen and skin prick test >3 mm and/or IgE to birch and timothy >0.35 kU/L were randomized to either ILIT, with three doses of 0.1 mL of birch pollen and 5‐grass pollen allergen extracts on aluminium hydroxide (10,000 SQ‐U/ml; ALK‐Abelló) or placebo using ultrasound‐guided intralymphatic injections at monthly intervals. Daily combined symptom medical score and rhinoconjunctivitis total symptom score were recorded during the peak pollen seasons the year before and after treatment. Rhinoconjunctivitis total symptom score, medication score and rhinoconjunctivitis quality of life questionnaire were recorded annually starting 2 years after treatment. Circulating proportions of T helper cell subsets and allergen‐induced cytokine and chemokine production were analysed using flow cytometry and ELISA.

**Results:**

There were no differences between the groups related to daily combined symptom medical score the year before and after treatment. Two years after ILIT (after unblinding), the actively treated group reported significantly fewer symptoms, lower medication use and improved quality of life than did the placebo group. After the pollen seasons the year after ILIT, T regulatory cell frequencies and grass‐induced IFN‐γ levels increased only in the actively treated group.

**Conclusion:**

In this randomized controlled trial, ILIT with birch and grass pollen extract was safe and accompanied by immunological changes. Further studies are required to confirm or refute the efficacy of the treatment.

## Graphical abstract

Intralymphatic immunotherapy with birch and grass pollen extracts in allergic rhinitis was safe and convenient but ineffective during first‐year randomized double‐blind clinical trial. In an open follow‐up 2 years after treatment, symptoms, medication intake and quality of life were significantly improved in the active treated group. T regulatory cell frequencies and grass‐induced IFN‐γ levels increased only in the actively treated group after the pollen seasons the year after ILIT.

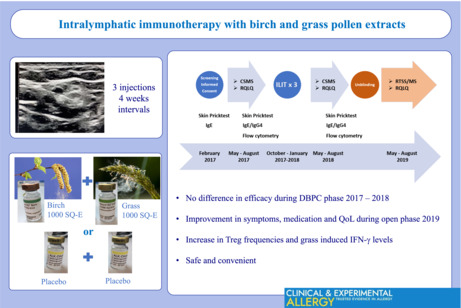

**Key messages**

- Intralymphatic immunotherapy in allergic rhinitis was safe and convenient but ineffective during first‐year DBPC trial.
- In an open follow‐up 2 years after ILIT, symptoms, medication intake and QoL were improved.
- Intralymphatic immunotherapy may be associated with immunomodulatory responses mediated by T regulatory cells.

## 1 INTRODUCTION

Allergen immunotherapy for allergic rhinitis is the only known intervention for inducing tolerance to an allergen.^1^ Current methods include subcutaneous immunotherapy (SCIT) and sublingual immunotherapy (SLIT), which are associated with the modulation of innate immune responses such as lower local mast cell, basophil, eosinophil and circulating group 2 innate lymphoid cell frequencies, as well as changes in adaptive immune responses such as induced allergen‐specific blocking antibodies (IgG and IgA), immunoregulatory cytokines like IL‐10, and increased proportions of regulatory T and B cells.^2^, ^3^ In Sweden, Alutard (ALK‐Abelló, Hørsholm, Denmark) is mainly used for SCIT. It requires about 40 subcutaneous injections during an updosing phase of 7–15 weeks, followed by a maintenance phase of 3 years. SLIT requires patients to take tablets containing allergens daily for 3 years.^4^ A faster, more efficient, and safer means is required to induce tolerance in patients with severe allergic rhinitis.^5^, ^6^, ^7^ Since 2008, several studies have suggested that intralymphatic immunotherapy may be one such means.^8^, ^9^ Patients receiving three monthly ultrasound‐guided injections with allergen over 8 weeks show improvements in symptoms and quality of life despite lower use of medication.^10^, ^11^, ^12^, ^13^, ^14^, ^15^, ^16^, ^17^, ^18^, ^19^

We have reported clinical improvement in symptoms and quality of life and less need for medication in patients with allergic rhinitis due to birch and grass pollen after treatment with ILIT with birch or timothy extracts in an open pilot study.^20^ We found fewer T helper (Th) 2 cells, more T regulatory (Treg) cells and IL‐10 secretion after ILIT. We have also reported a study of 72 patients with the same condition who were randomized to treatment with either birch or grass pollen extract and placebo or both birch and grass pollen extracts.^21^ Surprisingly, we found that ILIT with one or two allergens rendered similar clinical responses during the subsequent three birch and grass pollen seasons. We observed increased Treg cell frequencies 3 years after completed ILIT and activated Treg (aTreg) cells showed a similar pattern.^21^ After ILIT, only slightly less specific IgE was reported, with no changes in IgG4 levels and skin prick test reactivity.^21^ This is in line with other ILIT studies, where effects on T cells and their cytokines have primarily been observed.^13^, ^16^, ^18^, ^22^ However, our previous study lacked a true placebo group as all the participants received at least one active substance.^21^ Thus, we performed a randomized double‐blind placebo‐controlled clinical trial to explore the clinical and immunological impact of ILIT with birch and grass.

## 2 METHODS

### 2.1 Study design

**FIGURE 1:**
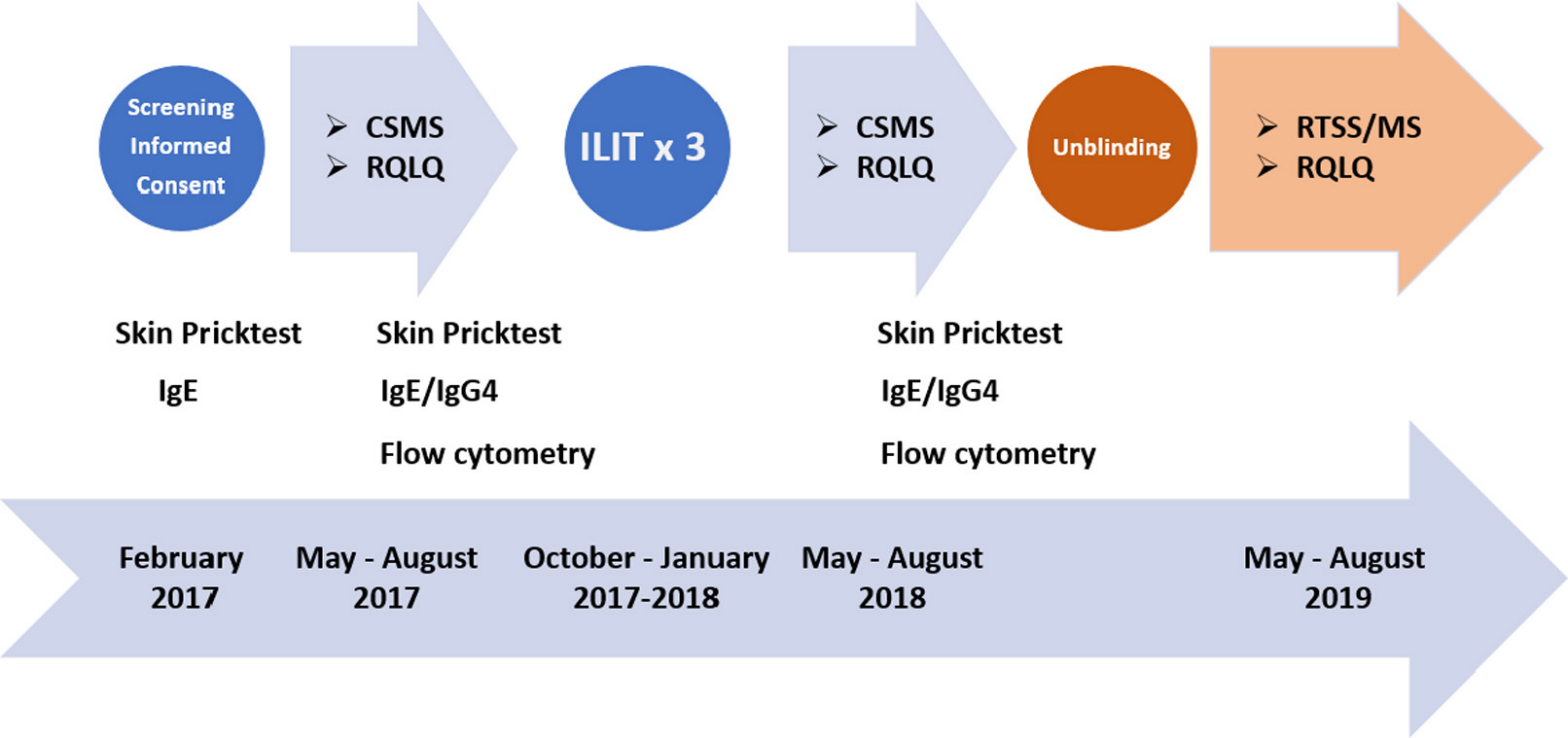
Combined symptom medical score (CSMS), and RQLQ, rhinoconjuntivitis quality of life questionnaire, were assessed during the double‐blind phase of the study, that is the birch and grass pollen seasons before (2017) and after ILIT (2018). The study was then unblinded and continued as an open study. Participants estimated their symptoms as RTSS, rhinoconjunctivitis total symptom score, medication as MS, medication score see supplement, and RQLQ after the birch grass pollen season.

The present study is a randomized double‐blind placebo‐controlled clinical trial in 37 patients with rhinitis due to sensitization to birch and grass pollen allergens. It was double‐blind during the first 2 years (2017–2018) and unblinded after evaluating the pollen seasons in 2018, the year after treatment according to the protocol. It continues as an open study until 2025. In this paper, we report on the first year of the open study as well (2019) (Figure 1).

### 2.2 Study population

**FIGURE 2:**
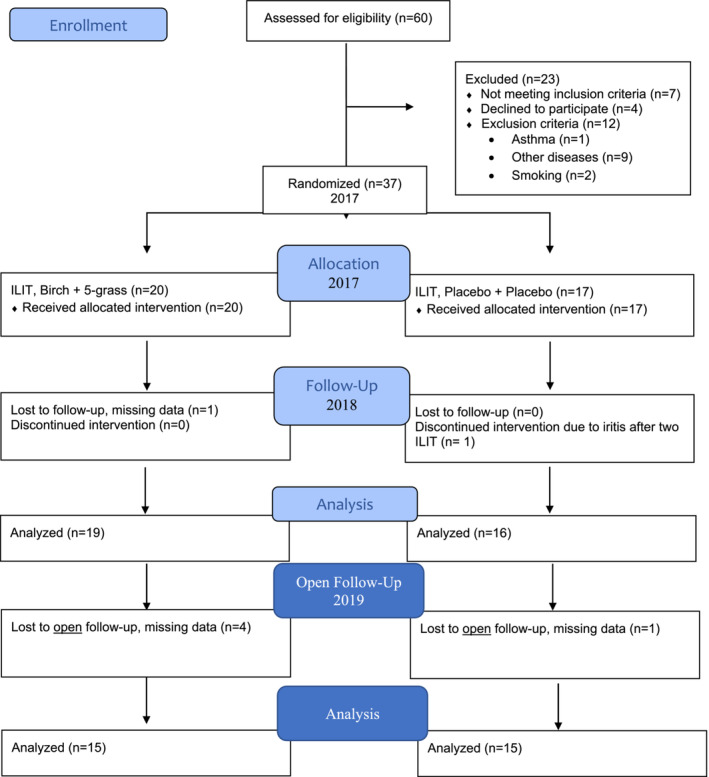
Flow chart.

The study was designed in 2015. In a previous study,^21^ we found significant improvement in RTSS (39–42%) and MS (48–49%) scores 3 years after treatment with ILIT using birch and grass or either, and a placebo. Based on our results (RTSS and MS), we calculated the power for finding a 50% difference at 0.05 with a power of 90% and confirmed that we needed 44 patients. As the EAACI had agreed to use a combined symptom score in 2014,^23^ we used the CSMS according to EAACI. However, as reference values with the CSMS have not been published in other studies, the power was calculated from our earlier RTSS and MS data. Sixty subjects were assessed for eligibility. Twenty‐three were excluded, as they did not meet inclusion criteria, declined to participate, or displayed exclusion criteria. Thus, study subjects were randomized to active ILIT with birch and grass pollen extracts or placebo (Figure 2).

Participants had seasonal allergic symptoms to birch and grass pollen, and a rhinoconjunctivitis total symptom score (RTSS)^24^ >7, with skin prick test >3 mm and/or IgE to birch and timothy >0.35 kU/L. Exclusion criteria were perennial pulmonary disease, <75% of predicted forced expiratory volume at the end of the first second in percent of predicted value (FEV1),^25^, ^26^ use of more than 800 μg inhaled budesonide (or equivalent) per day, pregnancy, severe arterial hypertension, autoimmunity, cardiovascular, hepatic, renal, upper airway or metabolic disease, mental incapability, alcohol abuse, smoking, medication affecting immune response or beta‐blockers. Baseline characteristics by treatment groups are presented in Table 1.

**TABLE 1:** Baseline characteristics by treatment groups.

	Birch + 5‐grass	placebo + placebo
*n*	20	17
Female	4 (20%)	6 (35%)
Mean age at study start	39.4 ± 11.8	28.6 ± 10.3
Min/max age at study start	21/54	19/51
IgE Birch (kU/L)	14.50	11.35
Median (IQR)	(7.42–56.13)	(3.28–40.40)
IgE Timothy (kU/L)	12.50	8.750
Median (IQR)	(4.72–26.0)	(1.22–19.10)
SPT Birch (mm)	6.0	6.5
Median (IQR)	(4.5–7.0)	(5.5–7.9)
SPT Timothy (mm)	6.0	6.0
Median (IQR)	(4.5–7.5)	(4.0–9.0)
Other sensitizations^a^	2.7 ± 1.8	3.5 ± 2.6
RTSS birch previous year	12.3 ± 2.7	12.7 ± 3.1
RTSS grass previous year	12.8 ± 2.4	13.3 ± 2.4
FEV1%	92.9 ± 9.8	92.1 ± 10.8
FENO ppb	20.6 ± 22.3	18.8 ± 8.7

Abbreviations: FENO‐ppb, fraction of exhaled nitric oxide in parts per billion; FEV1%, forced expiratory volume at the end of the first second in percent of predicted value; RTSS, Rhinoconjunctivitis total symptom score; SPT, skin prick test.

^a^ Number of other positive skin prick tests with mugwort, cat, dog, horse, *Dermatophagoides pteronyssinus, Dermatophagoides farinae, Cladosporium, Alternaria* and *Aspergillus* extracts (Soluprick SQ, ALK‐Abelló).

### 2.3 Intralymphatic immunotherapy

The randomization was done by Forum Östergötland, Sweden. An unblinded nurse prepared and marked each syringe with a label providing randomization number, injection number and injection site. ILIT was performed with three doses of 1000 SQ‐U, that is 0.1 mL of birch pollen allergen on aluminium hydroxide (10,000 SQ‐U/ml; ALK‐Abelló) and 0.1 mL of 5‐grass pollen allergen on aluminium hydroxide (10,000 SQ‐U/ml; ALK‐Abelló), given in the right and left groin at four‐week intervals or 0.1 mL placebo diluent (ALK‐Abelló), one in each groin (see Appendix S1). The grass extract (5‐grass) is a mix of equal SQ‐U of *Alopecurus pratensis* (meadow foxtale), *Dactylis glomerata* (cock's foot), *Festuca pratensis* (meadow fescue), *Lolium perenne* (English ryegrass) and *Phleum pratense* (timothy). Histamine‐1 blocker desloratadine 5 mg was given 20 min prior to the injections.

### 2.4 Pollen seasons

Birch and grass pollen seasons were defined according to EAACI recommendations.^27^ Daily birch and grass pollen counts were obtained from the Palynological laboratory, Swedish Museum of Natural History, from the measuring station in Norrköping, approximately 40 km from Linköping, Sweden (see Appendix S2).

### 2.5 Primary outcome measures

Symptoms and medication evaluated as recommended by EAACI using the CSMS^23^ are explained in detail in Appendix S1. The CSMS was answered by the patients daily through an inhouse application for smartphones or other digital platforms during the birch and grass pollen seasons the year before treatment in 2017 and the year after (2018). As it is a burden for the patients to fill in questionnaires daily, we did not require them to do so for more than 2 years. Effects on quality of life were measured for the season before treatment and after the following pollen seasons using the RQLQ.^28^ The RQLQ was answered by the patients every second week during the 2017 and 2018 pollen seasons. Data from the CSMS and RQLQ were assessed during 1 week of each peak pollen seasons in 2017 and 2018 for birch and grass, respectively.

### 2.6 Secondary outcome measures

The RTSS^24^ and MS from the Swedish Association for Allergy 2011 (see Appendix S3) were assessed separately after the birch pollen (approximately June 1st) and grass pollen seasons (approximately August 1st) after the study was unblinded in 2019, the second season after treatment. Skin prick test reactivity (Soluprick SQ Birch and Timothy; ALK‐Abelló), allergen‐specific IgE and allergen‐specific IgG4 levels were analysed (ImmunoCAP; ThermoFisher) after the season before ILIT, 2017, and 1 year after ILIT, 2018, before unblinding.

### 2.7 Safety assessment

Safety was assessed as the recording of adverse events from the time of the first injection to 1 year after the last injection. A research nurse called the patients to assess adverse events 2–5 days after each injection. The patients were questioned according to a special schema regarding pain, local reactions, symptoms from upper and lower respiratory systems, skin and general symptoms. Safety laboratory parameters, that is Hb, leucocytes, differential count of leucocytes, transaminases and creatinine, were assessed at screening, after the third ILIT injections and after the first pollen season following ILIT (see Appendix S4).

### 2.8 Immune laboratory methods

#### 2.8.1 Flow cytometry

Flow cytometry was used to analyse peripheral Th cell populations before ILIT and 1 year after completed treatment before unblinding, as described previously.^20^, ^21^ Tregs were gated for the expression of FoxP3 in the CD25dim and CD4dimCD25high populations. Activated Treg cells were gated for CD45RA^−^FoxP3^++^, and resting Treg cells were gated for CD45RA^+^FoxP3, as described previously^20^, ^21^ and in Appendix S1.

### 2.9 Cell stimulation and measurement of cytokines after allergen stimulation by ELISA

Cells were stimulated with allergens, as detailed in Appendix S1. The levels of IFN‐γ, IL‐5, IL‐10 and IL‐13 were determined in the supernatants using enzyme‐linked immunosorbent assay (ELISA), as described previously^29^ and in Appendix S1.

### 2.10 Statistics

Descriptive statistics for RQLQ, CSMS, IgE, IgG4 and skin prick test are presented as median value with IQR. The differences in RQLQ, RTSS, MS, CSMS values and data for T cells and cytokines were calculated and used for group comparison. As the data were not distributed normally, non‐parametric tests were used. The Mann–Whitney *U*‐test for continuous variables was used to compare the two treatment groups before and after treatment. Paired comparisons were calculated with Wilcoxon signed rank test. The clinical statistics were calculated with GraphPad Prism 8.0.1 (GraphPad software, Inc., La Jolla, CA, USA), with calculations for the immunological data performed in GraphPad Prism 9.3.1. *p‐*values <.05 were considered statistically significant.

### 2.11 Ethical considerations

This EudraCT (2016–003369‐24) study was approved by the Regional Ethics Committee in Linköping, Sweden (EPN Dnr 2016/400–31). Informed signed consent was obtained from the participants before inclusion.

## 3 RESULTS

### 3.1 Symptoms, medication and health‐related quality of life during birch pollen seasons

**FIGURE 3:**
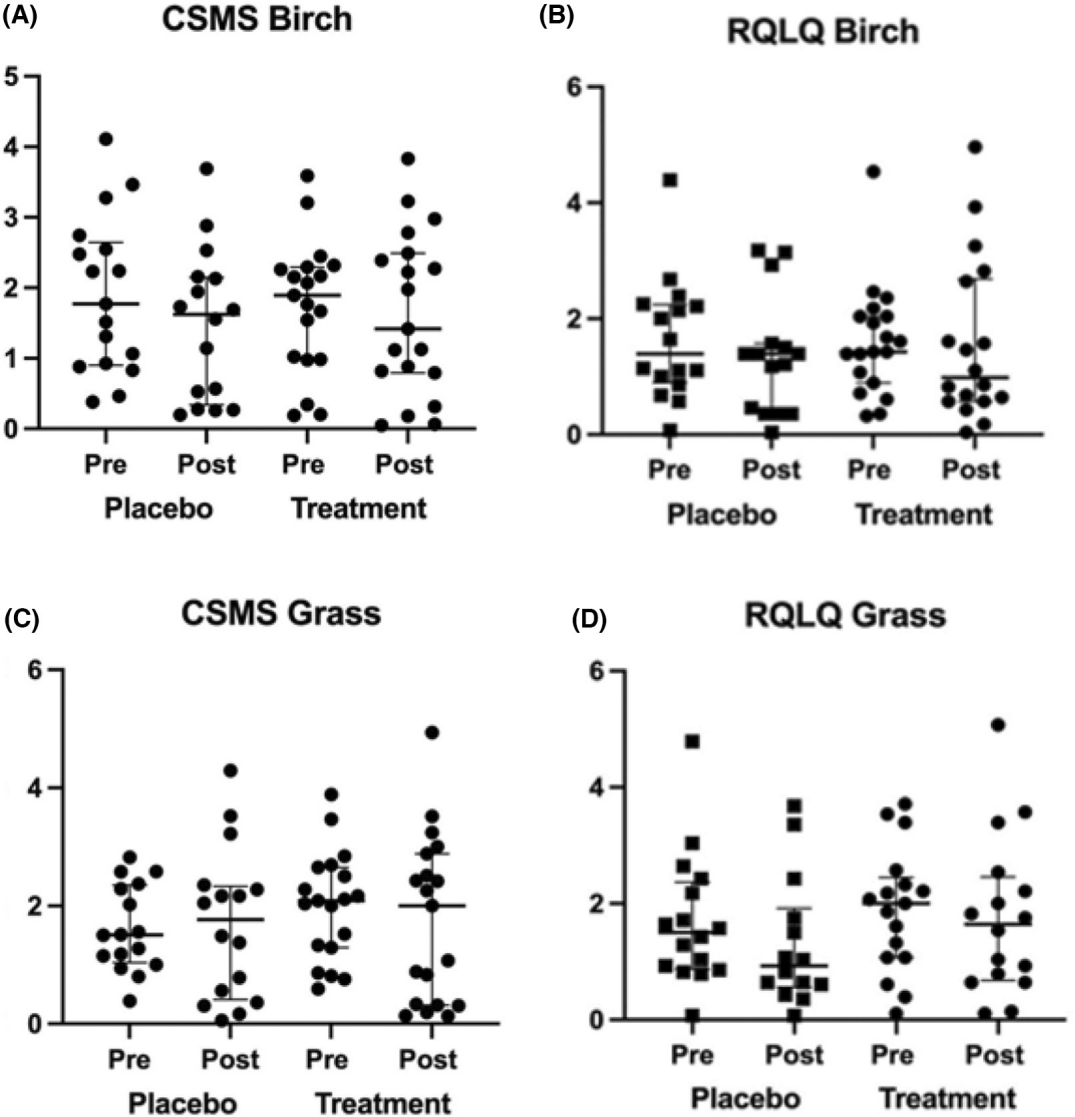
CSMS (0‐6), combined symptom medical score and RQLQ (0‐6), rhinoqonjuntivitis quality of life questionaire reported by the participants during the birch (A and B) and grass peak pollen seasons (C and D) pretreatment 2017 and post‐treatment 2018. The Mann‐Whitney U‐test for continuous variables was used to compare the two treatment groups before and after treatment. Paired comparisons were calculated with Wilcoxon Signed Rank test. There were no significant differences within or between the placebo group and the actively treated group before or after treatment. Combined symptom medical score and RQLQ during birch and grass pollen seasons pretreatment 2017 and post‐treatment 2018.

No significant differences in symptoms and medication measured as CSMS were reported during the peak birch pollen seasons in 2017 and 2018, within the active group, that is median level of 2.0 (IQR 0.83–2.5) in 2017, the year before ILIT, compared with 1.0 (IQR 0.17–2.67) in 2018, the year after ILIT. This was also true for the placebo group, which returned a median level of 1.58 (IQR 0.94–2.58) in 2017, compared with a median level of 1.108 (IQR 0.27–2.42) in 2018. There were no significant differences between the groups in 2017 or 2018 (Figure 3A). Health‐related quality of life measured as RQLQ during the peak birch pollen seasons in 2017 and 2018 was not significantly different, between or within the active 2017 group: median 1.43 (IQR 0.89–2.04), 2018: median 2.40 (IQR 0.86–2.64) and the placebo 2017: median 1.39 (IQR 0.89–2.24), 2018: median 1.30 (IQR 0.35–3.18) group (Figure 3B).

**FIGURE 4:**
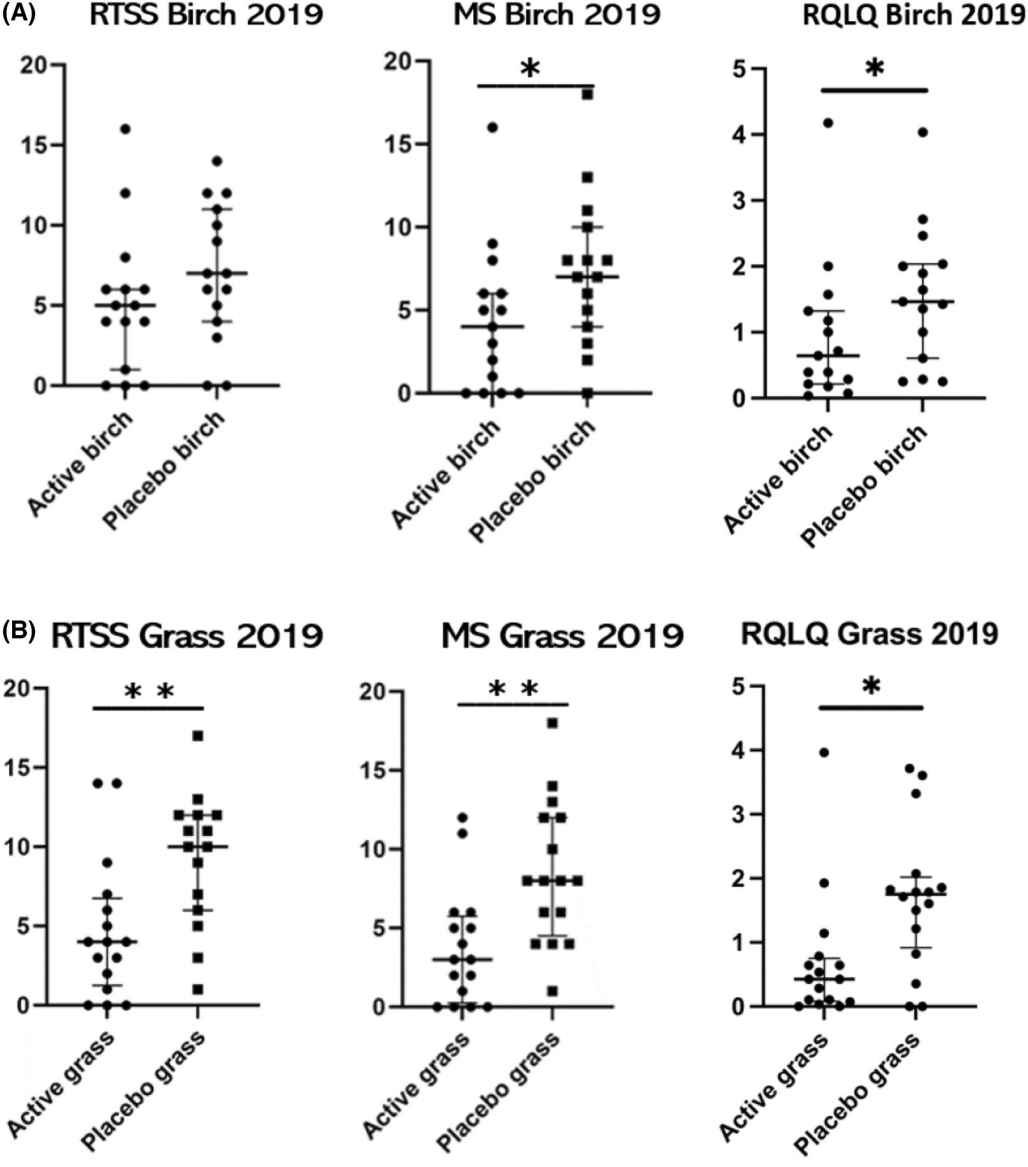
Symptoms measured as RTSS (0‐18) (rhinoconjunctivitis total symptom score), Medication measured as MS (0‐40) (medication score) and health‐related quality of life measured as RQLQ (0‐6) answered after the a) birch pollen season (approximately June 1st) 2019 and b) grass pollen season (approximately August 1st) 2019. For comparisons between the two treatment groups, Mann‐Whitney U‐test was used. *p < .05, *p < .01 Rhinoconjunctivitis total symptom score, MS and RQLQ birch (A) and grass pollen season (B) 2019.

In the open follow‐up 2 years after ILIT in 2019 (Figure 4A), symptoms after the birch pollen season measured by the RTSS were median 5.0 (IQR 1.0–6.0) in the active group and median 7.0 (IQR 4.0–11.0) in the placebo group (*p* = .17). However, medication measured by the MS was significantly lower in the active group than in the placebo group (*p* < .05). Moreover, the RQLQ during birch pollen season was significantly lower in the active group than in the placebo group (*p* < .05).

### 3.2 Symptoms, medication and health‐related quality of life during grass pollen seasons

No significant difference in the CSMS was observed in the placebo group between the peak grass pollen seasons before ILIT 2017 (1.50 (IQR 1.02–2.29)) and after ILIT 2018 (1.83 (IQR 0.33–2.70)) (Figure 3B). The CSMS in 2017 and 2018 were also similar in the active and placebo groups (2.00 (IQR 1.17–2.58) and 1.50 (IQR 0.25–2.83), respectively). No significant differences were found in the CSMS between the active and the placebo group in 2017 or 2018 (Figure 3C). Likewise, no significant differences were found in health‐related quality of life measured using the RQLQ during the grass pollen seasons in 2017 and 2018, between or within the active group in 2017: median 1.86 (IQR 0.6–2.32), in 2018: median 1.04 (IQR 0.14–2.21), and the placebo group in 2017: median 1.50 (IQR 0.87–2.37), in 2018: median 0.73 (IQR 0.37–1.69) (Figure 3D).

**FIGURE 5:**
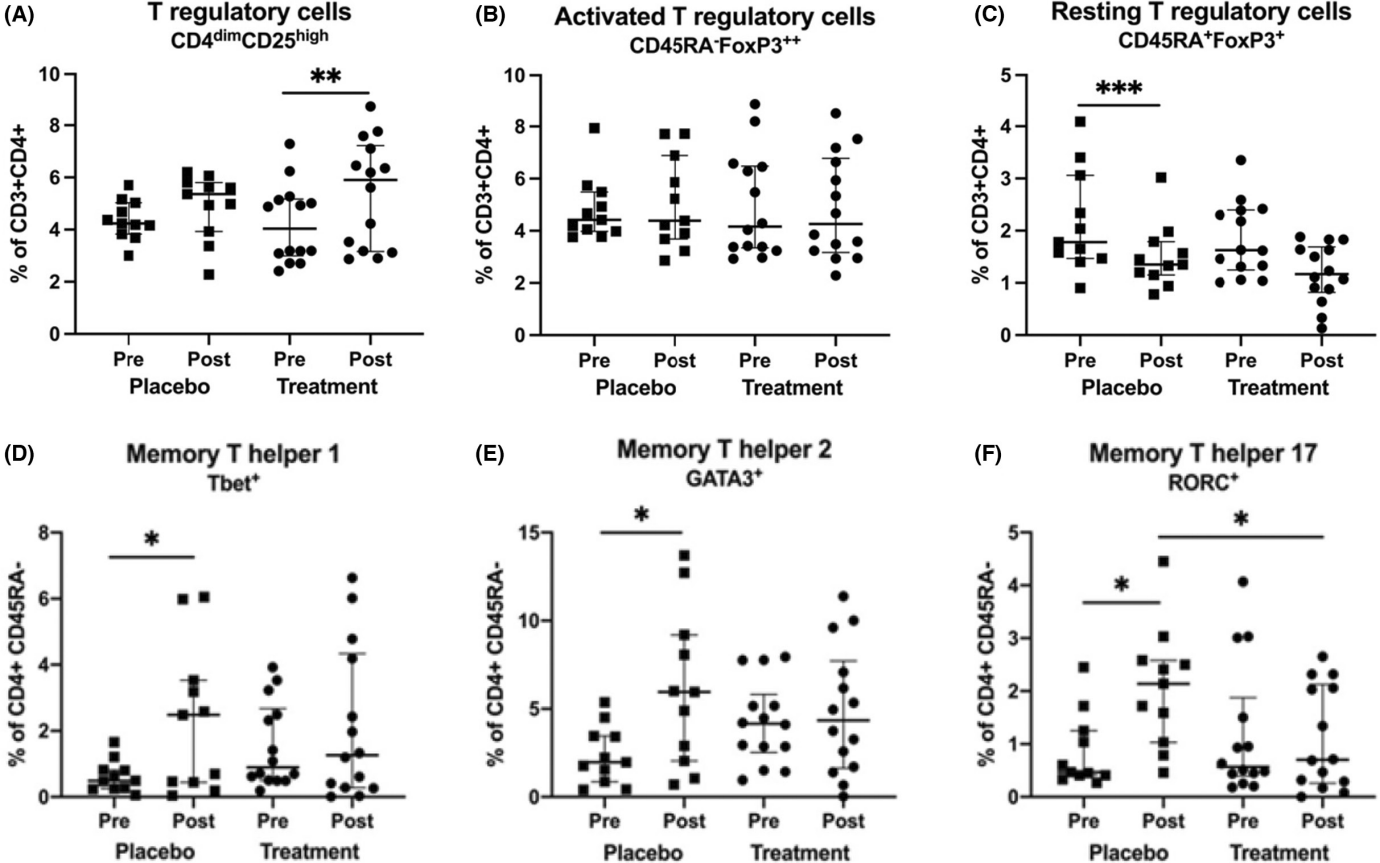
Proportion (%) of T regulatory (Treg) cells defined as CD4^dim^CD25^high^ (A), activated Treg cells defined as CD45RA^−^FoxP3^++^ (B), resting Treg cells defined as CD45RA^+^FoxP3^+^ (C) in the CD3^+^CD4^+^ population, different memory T helper (Th) cells, Th1 defined as CD4^+^CD45RA^−^Tbet^+^ (D), Th2 defined as CD4^+^CD45RA^−^GATA3^+^ (E) and Th17 defined as CD4^+^CD45RA^−^RORC^+^ (F) after intralymphatic immunotherapy with birch and 5‐grass allergen (*n* = 14, circles) or placebo (*n* = 11, squares). Blood samples were collected at randomization (pre) and 1 year after the intervention had finished (post). Groups were compared using Mann–Whitney *U*‐test and Wilcoxon matched‐pairs signed rank test was used to compare differences within each treatment group, *p < 0.05, **p < 0.01, ***p < 0.001. The data is presented as median and interquartile range (25th and 75th percentile values).

In the open follow‐up in 2019 (Figure 5B), the RTSS after the grass pollen season was significantly lower in the active group, median 4.0 (IQR 1.25–6.75), than in the placebo group, median 10.0 (IQR 6.0–12.0) (*p* < .01). Furthermore, the MS was also significantly lower, median 3.0 (IQR 0.25–5.75) in the active group than in the placebo group, median 8.0 (IQR 4.5–12.0) (*p* < .01). Moreover, the RQLQ during the grass pollen season was lower in the active group, median 0.43 (IQR 0.08–0.75), than in the placebo group, median 1.7 (IQR 0.92–2.02) (*p* < .05) (Figure 4B).

### 3.3 Safety

The 37 patients in the study received a total of 220 injections. Common adverse events after treatment were local itch, redness, pain and swelling at the injection site (7.3% in the active group and 2.7% in the placebo group) and tiredness (5.0% in the active group and 4.5% in the placebo group). They were all judged to be mild or moderate (Appendix S5). One patient reported a recurrence of iritis after the second injection. The patient had had iritis 4 years previously but had not disclosed this at the first visit. The patient was excluded from the study. However, when unblinded, the data showed that the patient had received a placebo. One patient reported severe joint pain 2 weeks after the first injection and was referred to primary care. No signs or blood tests indicated rheumatic disease. The patient received the additional injections without experiencing joint pain and had also received a placebo.

### 3.4 IgE, IgG4 and skin prick tests

Levels of IgE antibodies against birch and timothy increased significantly after ILIT treatment between 2017 and 2018 in the active group, but not in the placebo group (Table 2). However, there were no significant differences between the active and placebo groups in 2018, the year after treatment. Nor were there significant differences in IgG4 levels between the two groups in 2018. Skin prick test reactivity for timothy, but not for birch, increased significantly in the active group between 2017 and 2018, but no significant differences were evident in the size of skin prick tests between the treatment groups in 2018 (Table 2). IgE, IgG4 and skin prick tests were not assessed after unblinding in 2018.

**TABLE 2:** IgE and IgG4 antibody levels and skin prick tests in the active and placebo groups.

	2017 Active	2017 Placebo	2018 Active	2018 Placebo
IgE Birch	14.50	11.35	23.0	13.90
(kU/L)	(7.42–56.13)	(3.28–40.40)	(7.88–88.28)**	(3.7–29.65)
IgE Timothy	12.50	8.750	13.0	7.30.0
(kU/L)	(4.72–26.0)	(1.22–19.10)	(6.30–41.0)*	(1.20–28.70)
IgG4 Birch	0.21	0.24	0.42	0.48
(mg/ml)	(0.10–0.44)	(0.13–0.67)	(0.20–0.76)	(0.22–0.84)**
IgG4 Timothy	0.20	0.19	0.36	0.33
(mg/ml)	(0.12–0.54)	(0.01–0.33)	(0.24–0.60)	(0.19–0.56)***
SPT Birch	6.0	6.5	6.5	6.5
(mm)	(4.5–7.0)	(5.5–7.9)	(6.0–8.0)	(5.5–7.9)
SPT Timothy	6.0	6.0	8.0	7.0
(mm)	(4.5–7.5)	(4.0–9.0)	(7.5–9.0)*	(4.9–9.0)

*Note*: All values are given as median and IQR (25%–75%).

Abbreviations: IgE, immunoglobulin E; IgG4, immunoglobulin G4; SPT, skin prick test.

* indicates *p*‐values for changes within groups in 2017–2018 calculated by Wilcoxon matched pair signed rank test * *p* < .05, **p < .01, ***p < .001. No significant changes were found between groups (active – placebo) when calculated by Mann–Whitney *U*‐test.

### 3.5 Circulating T helper cell subsets

The proportion of Treg (CD4^dim^CD25^high^) cells increased over time in the group that received active ILIT, but not in the placebo group (Figure 5A, *p* < .01). Activated Treg (CD45RA^−^FoxP3^++^) cell frequencies did not change in any group (Figure 5B). However, proportions of resting Treg (CD45RA^+^FoxP3^+^) cells decreased over time in the placebo group (Figure 5C, *p* < .001), as they did in the group that received active ILIT (*p* = .058). To investigate the effect of ILIT on Th1/Th2/Th17 cells, the proportion of CD4 + CD45RA memory cells expressing the corresponding transcription factors Tbet, GATA3 and RORC was measured using flow cytometry before and 1 year after ILIT. Whereas the proportion of all Th subpopulations increased over time in the placebo group (Figure 5D–F, *p* < .05), this was not observed in the group that received the active treatment. In addition, lower levels of Th17 (CD4^+^CD45RA^−^RORC^+^) cells were observed in the active than in the placebo group after ILIT (Figure 5F, *p* < .05).

### 3.6 Allergen‐induced cytokine production

**FIGURE 6:**
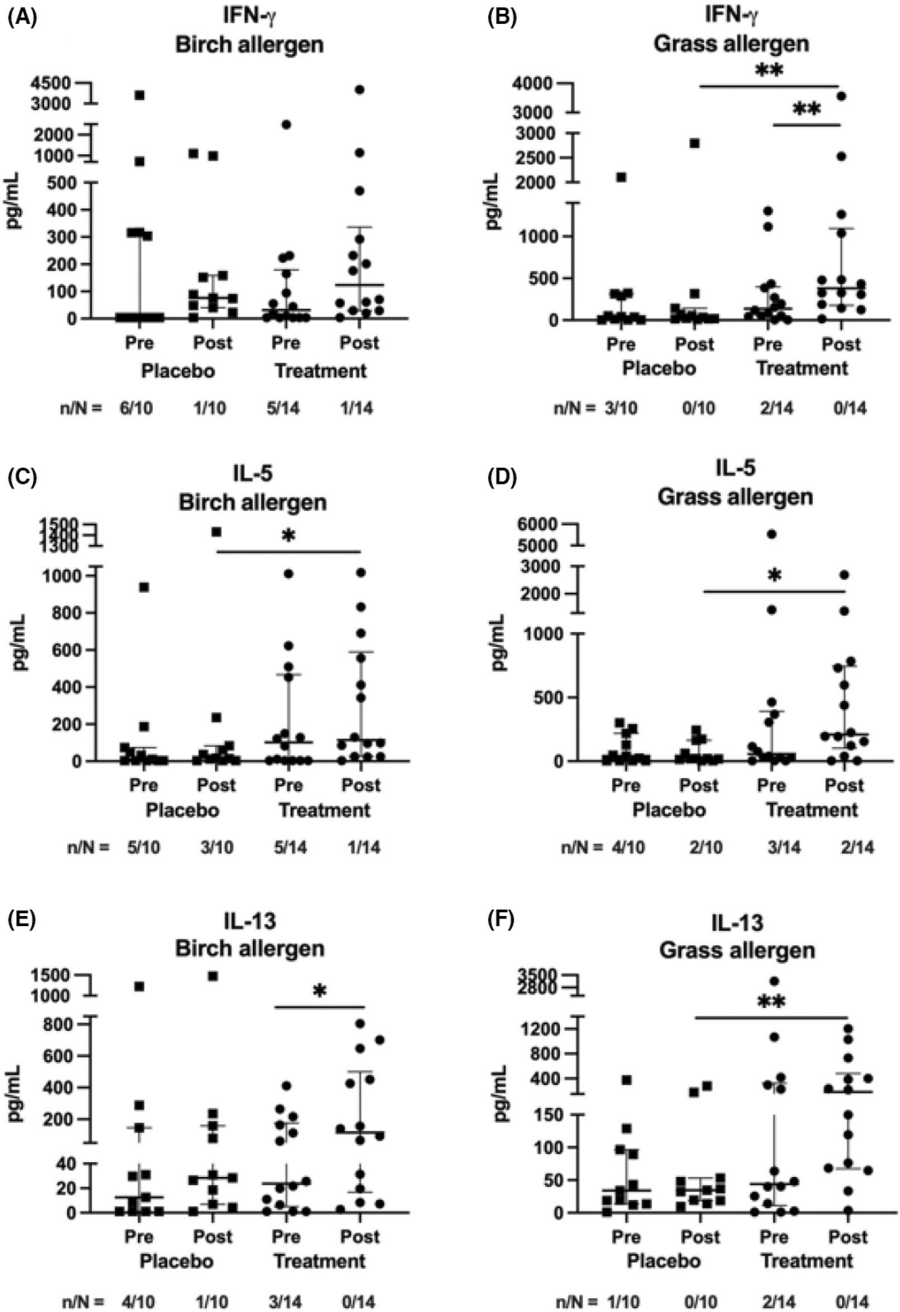
T helper cell‐associated cytokines, IFN‐γ (A, B), IL‐5 (C, D) and IL‐13 (E, F) were measured in supernatants from PBMCs stimulated with birch or grass allergen for 6 days after intralymphatic immunotherapy with birch and 5‐grass allergen (*n* = 14, circles) or placebo (*n* = 11, squares). Blood samples were collected at randomization (pre) and 1 year after the intervention had finished (post). Groups were compared using Mann–Whitney *U*‐test and Wilcoxon matched‐pairs signed rank test was used to compare differences within each treatment group, * *p* < 0.05, **p < 0.01. The data are presented as median and interquartile range (25th and 75th percentile values). The number of individuals below the detection limit (*n*) and the total number of individuals in the analysis (*N*).

Allergen‐induced IFN‐γ response remained unchanged when stimulated with birch pollen allergens (Figure 6A). However, grass‐induced IFN‐γ levels increased after ILIT within the active group, but not in the placebo group (Figure 6B, *p* < .01). IL‐5 levels did not change within the groups, but the IL‐5 levels were higher in the active group than in the placebo group before and after treatment (Figures 6C,D, *p* < .05). Birch allergen‐induced IL‐13 levels increased in the active group but not in the placebo group after completed treatment (Figure 6E, *p* < .05). Grass allergen‐induced IL‐13 was higher after treatment in the active group than in the placebo group (Figure 6F, *p* < .01), but no changes were observed within the groups before and after treatment (Figure 6F).

IL‐10 levels after 24 h could not be determined in many of the samples as the cytokine levels were under the detection limit. For the 6‐day stimulation, levels were similar in both groups (data not shown).

## 4 DISCUSSION

In this double‐blind randomized treatment with ILIT using birch and grass allergens, we were unable to find differences in the CSMS and RQLQ during the first year of follow‐up. However, immunological changes related to treatment with immunotherapy, that is increasing Treg cell frequencies^2^, ^3^ after birch and grass stimulation, were observed 1 year after active ILIT. Moreover, grass‐induced IFN‐γ levels increased after active ILIT. In the open follow‐up 2 years after ILIT, we found reductions in symptoms, less need for medication and improved quality of life. IgE antibodies, IgG4 antibodies and skin prick test reactivity did not correlate with clinical improvement during the first year after treatment.

We were unable to show improvement resulting from ILIT treatment measured by the CSMS or RQLQ in the first pollen seasons after the treatment. This may be explained by an extremely low birch pollen count in southeastern Sweden the season before treatment followed by a normal count the year after (see Appendix S2). During the second season after treatment, in the open follow‐up in 2019, we found a clear positive clinical effect of ILIT after treatment after unblinding, particularly for grass. During the extreme birch pollen season of 2019, the differences between the actively treated and placebo‐treated groups were significant, with less medication intake and better quality of life in the actively treated group. However, significant improvement of symptoms defined by the RTSS was only found for grass.

Whereas the low pollen counts may explain the lack of efficacy in the first year, particularly for birch, it is also possible that the trial failed its primary endpoint because the treatment was ineffective. Moreover, the results during the second year might be explained by a clinical trial effect, the open design and the placebo effect. However, the immunological changes resulting from immunotherapy^2^, ^3^ after the first year preceding the clinical results the second year may suggest otherwise. Furthermore, these results corroborate those from previous studies. Hellkvist et al published in 2018 an RDBPC ILIT trial comparing birch and 5‐grass with placebo, similar to the current study, with 24 in the active treatment group and 27 in the placebo group. They reported that symptoms were significantly reduced (28%) in the active group and non‐significantly (12%) in the placebo group. There was no significant difference between the groups, and the VAS overall improvement score between the groups did not differ significantly.^16^ However, in an open follow‐up of the study, the CSMS was lower during birch and grass pollen seasons in the actively treated group than in the placebo group 5–6 years after treatment.^30^

In the current study, no severe adverse events (AEs) have been reported. Only few local reactions such as itching, swelling or redness at the injection site, and several mild or moderate systemic reactions were reported by the participants. This is in line with previous ILIT studies. In a previous study, anaphylactic reactions occurred in a study of high‐dose ILIT with up to 5000 SQ‐U of allergen.^18^ Mild reactions are also common after SCIT.^31^, ^32^ In a large study that analysed adverse events in 1700 patients who had received SCIT, systemic AEs were reported by 3.3% of the patients and in 1.56/1000 injections.^32^ Oedema and pruritus at the injection site, flush, urticaria, wheezing, dyspnoea, eye pruritus, headache and abdominal pain are common (1%–10%) or very common (>10%) with SCIT, whereas oral pruritus, oral oedema, rhinitis, headache, ear pruritus, throat irritation, asthma, abdominal pain, urticaria and fatigue are common or very common with SLIT.^33^ ILIT seems to cause AEs at a similar rate to SCIT per injection, but because ILIT only needs three injections, the AEs can be reduced by up to 90% compared with SCIT.

In a previous study^16^ of ILIT with both birch and grass pollen extracts, a significant but modest increase was observed for grass‐specific IgG4 (mean 0.18–0.3 mg/L), but not for birch‐specific IgG4. SPT reactivity and allergen‐specific IgE levels were unchanged 6–9 months after treatment, which is similar to our current results. The levels of specific IgE against birch and timothy, and skin prick tests did not change after active treatment, as observed in our previous study^21^ and other ILIT studies.^11^, ^14^, ^16^, ^20^, ^22^ Additionally, the levels of IgG4 to birch and timothy were similar in both groups before and after the treatment. In previous ILIT studies, only moderate changes in allergen‐specific IgG4 have been reported.^11^, ^14^, ^16^, ^20^, ^22^ Different results regarding IgG4 levels in SCIT, SLIT and ILIT may depend on sample timing, dose and the administration route of the allergen.

We observed an increase in Treg cells (CD4^dim^CD25^high^) in the group that received active ILIT, whereas no changes occurred in the placebo group. In our previous ILIT study, no changes were observed at 1 year after completed ILIT for the Treg, and for aTreg cells only after 3 years.^21^ Th17 cells, which decreased after ILIT compared with the placebo group in the current study, were also in line with the results described previously.^21^ Furthermore, Hellkvist et al performed a fine needle aspiration from the lymph nodes before and 2–4 weeks after treatment in a subgroup (6 active and 6 placebo). The aspirates showed an increased proportion of memory T cells after treatment in the active group, and an increase in Treg cells in peripheral blood.^16^ This is in line with our results. As in our previous study,^21^ we saw an increase in allergen‐induced IL‐5 secretion in the group that received ILIT with birch allergen, possibly associated with early Th2 priming. Active ILIT was also related to an increase in birch induced IL‐13 production compared with pretreatment and placebo, also in line with early Th2 priming (Figure 6E). However, these changes may be counteracted by other regulatory changes, that is the increased Treg frequencies and grass‐induced IFN‐γ production observed after ILIT treatment.

If our results are robust, we need to understand the mechanisms behind the effect of ILIT. They may not be similar to SCIT or SLIT, although ILIT has a similar effect on the clinical symptoms of allergy. Allergen presentation in a lymph node may have more profound and different effects on the immunological reactions from allergen presentation, cellular activation and cytokine production. Although further research on this issue is needed, our immunological results, with increase Tregs and grass‐induced IFN‐γ production and no changes in allergen‐specific IgE or IgG4, point in that direction.

One limitation in the present study is that we could not identify any clinically beneficial differences between the groups using the daily CSMS the seasons before and after ILIT. Nonetheless, 2 years after treatment, in the open follow‐up, we found significant clinically relevant beneficial differences in the actively treated ILIT group with respect to symptoms, medication intake and quality of life related to allergic rhinitis. The study was double‐blinded during the first 2 years and unblinded according to the protocol after evaluating the pollen seasons the year after treatment. It will continue as an open study until 2025.

Allergic rhinitis entails considerable costs for society.^34^ SLIT and SCIT, although expensive, are cost‐effective.^35^, ^36^, ^37^ ILIT, with only three injections, may offer faster relief from symptoms, making it more cost‐effective for patients, health care and society.

In conclusion, although results were negative in the first year after treatment, this study adds to hitherto positive studies and suggests that ILIT is not only safe but may also be an effective way to treat pollen allergy. It is associated with immunological changes, including increased Treg frequencies and grass‐induced IFN‐γ production, and no changes in allergen‐specific IgE or IgG4. This may be an opportunity to make AIT more easily accessible to patients, at lower cost and less risk. Further dosing studies are required to establish the optimal dose with respect to efficacy and side‐effects.

## AUTHOR CONTRIBUTIONS

LA, UN, JB and MJ contributed to overall design of the study. LA and MJ contributed to overall procurement of funds. LA, PR and UN performed ILIT. LA, JB, UN, PR and KD contributed to clinical evaluation and sample collection. EA and MJ designed the laboratory experiments. EA, DG and LS performed the laboratory work. LA, EA and LS contributed to Statistical calculations. LA, EA, JB, UN, MJ and KD contributed to overall analysis of the data. LA contributed to drafting of the manuscript. All authors contributed to critical review of the manuscript.

## FUNDING INFORMATION

Region Östergötland, the Allergy Center in Linköping, the Medical Research Council of Southeast Sweden (FORSS), the Th Bergh Foundation and the Asthma and Allergy Association.

## CONFLICT OF INTEREST STATEMENT

LA has received honoraria as a speaker and/or adviser from AstraZeneca, Meda, Takeda, Teva, Boehringer Ingelheim, MSD, Sanofi, GSK, Airsonett and Novartis. JB has received honoraria as a speaker from ALK. EA, LS, UN, PR, DG, MJ and KD have no COIs.

## Supporting information


Appendix S1.

Appendix S2.

Appendix S3.

Appendix S4.

Appendix S5.

## Data Availability

The datasets used and/or analyzed during the current study are available from the corresponding author on reasonable request.
