# Supplementary material for: Intralymphatic immunotherapy with birch and grass pollen extracts. A randomized double‐blind placebo‐controlled clinical trial
Source: Clin Exp Allergy. 2023 Apr 4;53(8):809–20. doi: 10.1111/cea.14307 (PMC10947267; doi:10.1111/cea.14307)
Supplement: Supplementary file 1 — Appendix S1. [file CEA-53-809-s002.docx]

**S1. Methods**

**Combined symptom medical score (CSMS)**

Symptoms and medication were evaluated as recommended by EAACI using the Combined Symptom Medication Score (CSMS), a combination of (RTSS) (1) and a medical score (2) during the first season, and RTSS and medication, as medication score (MS) from the Swedish Association for Allergy 2011 (see the online repository) separately after the second season. To calculate the CSMS, the RTSS is evaluated with six domains ranging from 0–3. The sum of these domains is divided by 6 and converted to a daily symptom score with a maximum of 3. The daily medication score has a stepwise approach to medication. Antihistamine, local or peroral, will score 1, nasal steroids, with or without antihistamines local or per oral, will score 2, and oral corticosteroids will score 3. The CSMS is the sum of the daily RTSS divided by 6 and the daily medication score, with a maximum total of 6.

**Intralymphatic immunotherapy**

The ILIT was performed by three clinicians (LA, PR, UN). Ultrasound-led technology (Siemens Acuson Freestyle) was used whereby a lymph node was punctured with a 27G (0.4 x 40 mm) needle. The groin was cleaned with chlorhexidine/ethanol. A small amount of non-sterile ultrasonic gel (Aquasonic 100 Parker Laboratories, INC, Fairfield New Jersey) was applied to the ultrasound probe (L13-5), which was then covered with a condom (PROFIL/MAGIC, THE ORIGINAL, rfsu ce 0413), and the area being explored. After the injection, the probe was cleaned with isopropanol 45%.

**Measurement of cytokines after allergen stimulation by ELISA**

PBMCs were isolated by Ficoll gradient centrifugation as previously described (3, 4). Aliquots with 1 x 10^6^ cells were stimulated with birch or timothy Aquagen allergen extracts (ALK-Abelló) at 10,000 SQ-U/mL for 24 hours (IL-10) or 6 days at 37°C with 5% CO_2_ in AIM-V serum-free medium (Life Technologies AB, Täby, Sweden) with 20 μM β-mercaptoethanol (Sigma-Aldrich). The supernatants were collected after centrifugation at 400 x g for 5 min and stored at –70°C until further analysis.

The levels of IFN-γ, IL-5, IL-10 and IL-13 were determined using enzyme-linked immunosorbent assay (ELISA), as described previously (5) with the following antibody-pairs; anti-IFN-γ (clone NIB42, BD Pharmingen, San Jose, USA) and anti-IL-5 (clone JES-39D10, BD Pharmingen), anti-IL-10 (Clone B-S10, OriGene Technologies, Rockville, USA), PeliPair™ human IL-13 ELISA reagent set (Sanquin, Amsterdam, the Netherlands). Standard curve was mixed using human recombinant IFN-γ (285-IF-100, BioTechne, Abingdon, United Kingdom) and IL-5 (BD Pharmingen), IL-10 (Pelipair), IL-13 (PeliPair). Biotinylated detection antibodies mouse anti-human IFN-γ (clone 4S.B3, BD Pharmingen) and rat anti-human IL-5 (clone JES1-5A10, BD Pharmingen), mouse anti-human IL-10 (clone B-T10, Origene), IL-13 (Pelipair) were used. The plates were analyzed using an ELISA reader (450 nm, wavelength correction 540 nm, Molecular Devices Co., Sunnyvale, USA) and the results were obtained using Magellan 7.1 software for IL-10 (Tecan Trading AG, Switzerland) or SoftMax pro 7.1 for IFN-γ, IL-5 and IL-13 (Molecular Devices). The lower detection limit was 7.8 pg/mL for IFN-γ and IL-5, 4.7 pg/mL for IL-10 and 2 pg/mL for IL-13. For statistical calculations, undetectable samples were given the value of half the cut off. The control*,* i.e., medium only, values were subtracted from the values from the allergen-induced response.

**Flow cytometry**

Flow cytometry was used to analyze peripheral T helper cell populations in whole blood drawn in EDTA tubes at randomization, one and three years after treatment had finished. Within the lymphocyte population (based on FSC and SSC), the Th population was defined using PECy7-conjugated anti-CD4 (SK3) and APCCy7-conjugated anti-CD3 (SK7) antibodies (BD Biosciences, San Jose, CA, USA). Within the CD3+CD4+ population, the naïve and memory cell populations were determined using v450-conjugated anti-CD45RA (HI100) antibodies (BD Biosciences). Expression of cellular Th cell lineage markers was determined using PE-conjugated anti-GATA3 (TWAJ), eFlour 660-conjugated anti-Tbet (eBio4B10), PE-conjugated anti-RORC (AFKJS-9), PerCP-Cy5.5-conjugated anti-CD25 (M-A251) and FITC-conjugated anti-Foxp3 (PCH101) antibodies (eBiosciences, San Diego, CA, USA).The proportion of Th subsets was determined using the baseline of 1% positive cells in the CD45RA^+^ naïve (undifferentiated), and the same gate settings were then transferred to the CD45RA^-^ memory (differentiated) CD3^+^CD4^+^ Th cell population. Treg cells were defined as CD4^dim^CD25^hi^Foxp3^+^. Subtypes of Treg cells were subdivided into CD3^+^CD4^+^CD45RA^+/-^Foxp3^+/++^, i.e., resting and activated Tregs (6), respectively. Complete gating strategy below. Data were acquired on a BD FACS CANTO II and analyzed using Kaluza 1.2 software (Beckman Coulter).

**Gating strategy**


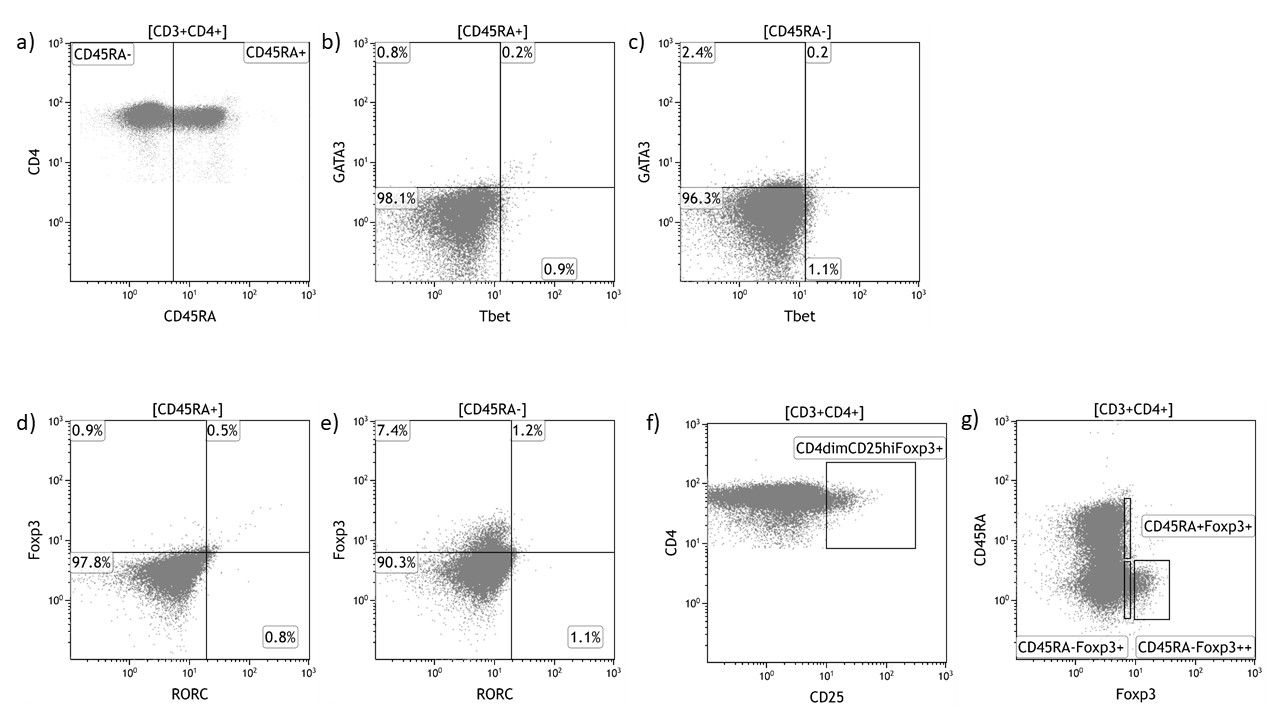
Gating strategy to identify T helper cell populations from flow cytometry analysis. A gate was set to isolate the lymphocyte population by measuring forward scatter (FSC) on the X axis (size) and side scatter (SSC) on the Y axis (granularity). To define T lymphocytes, a gate was set for CD3^+^CD4^+^ cells. The lymphocyte gate was also used to define CD3^+^CD4^+^ T helper (Th) cells. a) Naïve (CD45RA^+^) and memory (CD45RA^-^) Th cells were defined by their expression of CD45RA. b) To define Th1 and Th2 cells, the populations were gated on expression of the intracellularly expressed Th cell lineage markers, T-box expressed in T cells (Tbet), a transcription factor expressed by Th1, and GATA binding protein 3 (GATA3), a key transcription factor in Th2 cells. Cells in the naïve population were not expected to express the markers, and a gate was set in that population (maximum 1% and minimum 0.6% cells positive for the markers). In the memory population (c) The gate from the naïve population was used to define cells expressing Tbet and GATA3. d) and e) A similar strategy was used to define Th17 cells as RORC^+^ CD45RA^-^ cells. f) T regulatory cells were defined as CD4^dim^CD25^hi^Foxp3^+^ cells. g) In addition, two T regulatory subpopulations were isolated from the CD3^+^CD4^+^ Th cell population, depending on their expression of the transcription factor forkhead box P3 (FoxP3) and CD45RA. CD3^+^CD4^+^CD45RA^+/-^Foxp3^+/++^, i.e., resting and activated Tregs, respectively

1. Devillier P, Chassany O, Vicaut E, de Beaumont O, Robin B, Dreyfus JF, et al. The minimally important difference in the Rhinoconjunctivitis Total Symptom Score in grass-pollen-induced allergic rhinoconjunctivitis. Allergy. 2014;69(12):1689-95.

2. Pfaar O, Demoly P, Gerth van Wijk R, Bonini S, Bousquet J, Canonica GW, et al. Recommendations for the standardization of clinical outcomes used in allergen immunotherapy trials for allergic rhinoconjunctivitis: an EAACI Position Paper. Allergy. 2014;69(7):854-67.

3. Jenmalm MC, Bjorksten B, Macaubas C, Holt BJ, Smallacombe TB, Holt PG. Allergen-induced cytokine secretion in relation to atopic symptoms and immunoglobulin E and immunoglobulin G subclass antibody responses. Pediatric allergy and immunology : official publication of the European Society of Pediatric Allergy and Immunology. 1999;10(3):168-77.

4. Forsberg A, Abrahamsson TR, Bjorksten B, Jenmalm MC. Pre- and post-natal Lactobacillus reuteri supplementation decreases allergen responsiveness in infancy. Clinical and experimental allergy : journal of the British Society for Allergy and Clinical Immunology. 2013;43(4):434-42.

5. Bottcher MF, Bjurstrom J, Mai XM, Nilsson L, Jenmalm MC. Allergen-induced cytokine secretion in atopic and non-atopic asthmatic children. Pediatric allergy and immunology : official publication of the European Society of Pediatric Allergy and Immunology. 2003;14(5):345-50.

6. Miyara M, Yoshioka Y, Kitoh A, Shima T, Wing K, Niwa A, et al. Functional delineation and differentiation dynamics of human CD4+ T cells expressing the FoxP3 transcription factor. Immunity. 2009;30(6):899-911.
