# Supplementary material for: Intralymphatic immunotherapy with birch and grass pollen extracts. A randomized double‐blind placebo‐controlled clinical trial
Source: Clin Exp Allergy. 2023 Apr 4;53(8):809–20. doi: 10.1111/cea.14307 (PMC10947267; doi:10.1111/cea.14307)
Supplement: Supplementary file 2 — Appendix S2. [file CEA-53-809-s001.docx]

**Supplement/online repository**

**2A Birch pollen seasons 2017–2019**


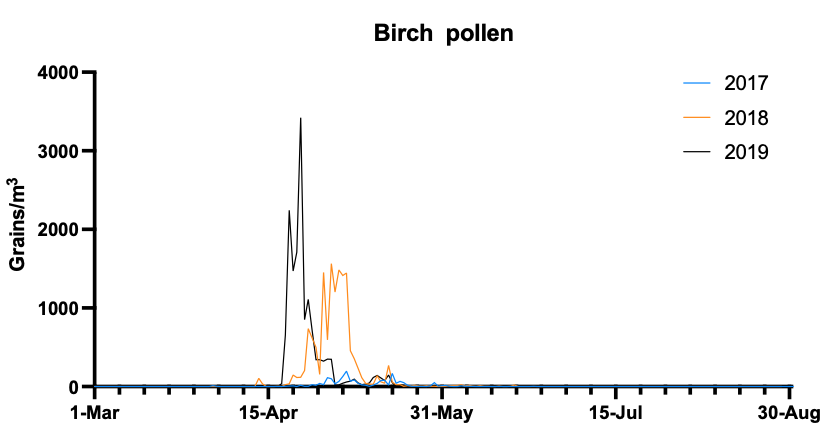


**2B Grass pollen seasons 2017–2019**


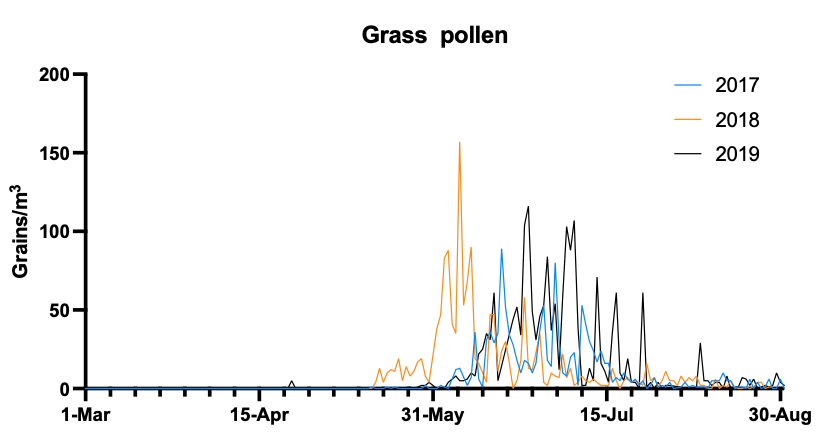


**Supplement 2A and B** Daily birch and grass pollen counts obtained from the Palynological laboratory, Swedish Museum of Natural History from the measuring station in Norrköping, approximately 40 km from Linköping, Sweden.
