# Supplementary material for: Intralymphatic immunotherapy with birch and grass pollen extracts. A randomized double‐blind placebo‐controlled clinical trial
Source: Clin Exp Allergy. 2023 Apr 4;53(8):809–20. doi: 10.1111/cea.14307 (PMC10947267; doi:10.1111/cea.14307)
Supplement: Supplementary file 3 — Appendix S3. [file CEA-53-809-s004.docx]

1. **Medication Score from the Swedish Association for Allergology 2011**

|  | **Never** | **Occasionally** | **Daily** |
| --- | --- | --- | --- |
| Oral antihistamine | 0p | 1p | 2p |
| Local treatment nose, except steroids | 0p | 1p | 2p |
| Inhaled bronchodilator | 0p | 1p | 1p |
| Nasal steroids | 0p | 2p | 4p |
| Other (i.e., montelukast, theophylline) | 0p | 2p | 4p |
| Peroral or ocular steroids | 0p | 4p | 8p |
| Steroid injection | 0p | 4p | - |
| Omalizumab | 0p | 8p | - |
