# Supplementary material for: Intralymphatic immunotherapy with birch and grass pollen extracts. A randomized double‐blind placebo‐controlled clinical trial
Source: Clin Exp Allergy. 2023 Apr 4;53(8):809–20. doi: 10.1111/cea.14307 (PMC10947267; doi:10.1111/cea.14307)
Supplement: Supplementary file 4 — Appendix S4. [file CEA-53-809-s003.docx]

## **Scheme of schedule procedures**

|  | **V1** | **V2** | **V3-5** | | | **V6** | |  | |
| --- | --- | --- | --- | --- | --- | --- | --- | --- | --- |
|  | **Screening** | **Randomization** | **Intervention** | | | **Follow-up** | | **Open follow-up** | |
|  | 02–04.17 | 08–09.17 | 10–11.17 | +4 w | +4w | 09–10.18 |  | | Every year until 2024 |
| Informed consent | x |  |  |  |  |  |  | |  |
| Phys. examination | x | x |  |  |  | x |  | |  |
| Blood pressure, pulse, PEF | x | x | x | x | x | x |  | |  |
| Safety tests^1^ | x | x |  |  |  | x |  | |  |
| Immunol. tests^2^ | x | x |  |  |  | x |  | |  |
| SPT | x | x |  |  |  | x |  | |  |
| Spirometry | x | x |  |  |  | x |  | |  |
| U-HCG (women) |  | x | x | x | x |  |  | |  |
| RQLQ, RTSS, MS^3^ | x | x |  |  |  | x |  | | x |
| CSMS^4^ | x |  |  |  |  | x |  | |  |
| AE |  | x | x | x | x | x |  | | x |
| Concom. med | x | x | x | x | x | x |  | |  |
| Diary teaching ^5^ | x |  |  |  | x | x |  | |  |
| ILIT |  |  | x | x | x |  |  | |  |
| Tel contact^6^ |  |  | x | x | x |  |  | |  |

Visit 1: Pre-ILIT, pre-season. Visit 2: pre-ILIT, post-season 2017. Visit 3; 7–35d post visit 2. Visit 4: 28-42d post visit 3. Visit 5: 28–42d post visit 4. Visit 6: post-season 2018

^1^ Hematology: leukocytes, leukocyte differentiation (neutrophil, eosinophils, basophils, lymphocytes) hemoglobin and platelets, creatinine, alanine aminotransferase, aspartate aminotransferase at visits 1, 2, and 6. Coagulation blood tests at visits 1 and 2.

^2^ Serology and immunological test.

^3^ RTSS = Rhinoconjunctivitis total symptom score, RQLQ = Rhinoconjunctivitis quality of life questionnaire and MS = Medical score were performed after the birch pollen season (approx. Jun 1^st^ 2019) and after the grass pollen season (approx. Aug 1^st^ 2019). RQLQ was answered by the patients every second week during the pollen seasons 2017 and 2018 and assessed for birch peak pollen season 2017: May1^st^ – May 7^th^. 2018: April 30^th^ – May 6^th^, and grass peak pollen season 2017: May 19^th^ – May 25^th^, 2018: May 11^th^ – May 17^th^.

^4^ CSMS = combined symptom medical score a daily in-house online form questionnaire. Birch peak pollen season 2017: May 1^st^ – May 14^th^, 2018: April 24^th^ – May 7^th^, grass peak pollen season 2017: June 19^th^ – July 3^rd^, 2018: June 2^nd^ – June 15^th^.

^5^ The diary had space for a description of the adverse events, AEs, since the last visit.

^6^ Two to five days after visit 3–5, telephone contact was made with patients concerning symptoms after the allergen injections.

All inclusion and exclusion criteria were checked at visits 1–5.

PEF: peak expiratory flow, SPT: skin prick test, U-HCG: urine human chorionic gonadotropin (only in females), ILIT: Intralymphatic immunotherapy.
