# Supplementary material for: Intralymphatic immunotherapy with birch and grass pollen extracts. A randomized double‐blind placebo‐controlled clinical trial
Source: Clin Exp Allergy. 2023 Apr 4;53(8):809–20. doi: 10.1111/cea.14307 (PMC10947267; doi:10.1111/cea.14307)
Supplement: Supplementary file 5 — Appendix S5. [file CEA-53-809-s005.docx]

**5**. Adverse events

| **Adverse event** |  | **Dose 1** | **Dose 2** | **Dose 3** |
| --- | --- | --- | --- | --- |
| Asthma/obstruction | Active  Placebo | 0  0 | 0  0 | 2  0 |
| Wheezy, without signs of bronchial obstruction | Active  Placebo | 1  2 | 1  2 | 0  1 |
| Injection site swelling | Active  Placebo | 4  1 | 3  0 | 3  0 |
| Pain at the injection site | Active  Placebo | 0  1 | 2  1 | 1  0 |
| Injection site redness | Active  Placebo | 3  0 | 2  1 | 2  1 |
| Injection site itch | Active  Placebo | 2  0 | 0  2 | 1  1 |
| Sneezing | Active  Placebo | 0  1 | 1  0 | 0  0 |
| Headache | Active  Placebo | 0  1 | 0  1 | 0  1 |
| Feeling hot | Active  Placebo | 1  1 | 0  0 | 0  0 |
| Food allergy | Active  Placebo | 0  1 | 0  0 | 0  0 |
| Eczema | Active  Placebo | 1  0 | 0  0 | 0  0 |
| Oral sore | Active  Placebo | 2  0 | 0  0 | 0  0 |
| Tiredness | Active  Placebo | 5  4 | 4  4 | 2  2 |
| Fever | Active  Placebo | 1  1 | 0  0 | 0  0 |
| Red eyes | Active  Placebo | 1  0 | 0  0 | 0  0 |
| Tenderness in jaw angle | Active  Placebo | 1  0 | 0  0 | 0  0 |
| Itch in knee folds | Active  Placebo | 0  1 | 0  0 | 0  0 |
| Joint pain | Active  Placebo | 0  1 | 0  0 | 0  0 |
| Oral allergy syndrome | Active  Placebo | 1  0 | 0  0 | 0  0 |
| Lumbago | Active  Placebo | 0  0 | 0  1 | 0  0 |
| Vasovagal reaction | Active  Placebo | 0  0 | 0  1 | 0  0 |
| Itch in throat | Active  Placebo | 0  0 | 0  1 | 0  0 |
| Subcutaneous hematoma | Active  Placebo | 0  0 | 1  0 | 0  0 |
| Vertigo | Active  Placebo | 0  1 | 0  0 | 0  0 |
| Migraine | Active  Placebo | 0  0 | 0  1 | 0  0 |
| Pneumonia | Active  Placebo | 0  0 | 0  1 | 0  0 |
| Iritis | Active  Placebo | 0  0 | 0  1 | 0  0 |
| Gastroenteritis | Active  Placebo | 0  1 | 0  0 | 0  0 |
| Abdominal pain/nausea | Active  Placebo | 0  1 | 0  0 | 0  0 |
| Total | Active  Placebo | 25  18 | 14  17 | 11  6 |
